# Prognostic significance of lymphocyte apoptosis markers (Annexin V, sFas, and sFasL) in sepsis: a retrospective clinical study

**DOI:** 10.3389/fcell.2026.1983746

**Published:** 2026-10-27

**Authors:** Yuxuanlin Liu, Jianqi Wu, Jinze Li

**Affiliations:** 1 Department of Clinical Laboratory, The First Affiliated Hospital of Jinzhou Medical University, Jinzhou, Liaoning Province, China; 2 Department of Clinical Laboratory, Liaoyang Second People’s Hospital, Liaoyang, China

**Keywords:** 28-day mortality, Annexin V, biomarker, immunosuppression, lymphocyte apoptosis, prognosis, programmed cell death, sepsis

## Abstract

**Background:**

Sepsis-associated lymphocyte apoptosis may contribute to immune dysfunction and adverse outcomes. Annexin V binding detects phosphatidylserine externalization, a general feature of early apoptosis that is not specific to Fas-mediated signalling; soluble Fas (sFas) and soluble Fas ligand (sFasL) are more directly related to the Fas/FasL system. Their combined prognostic associations in sepsis merit evaluation.

**Methods:**

In this reported single-centre observational cohort, 218 adults with Sepsis-3-defined sepsis were included. Annexin V positivity was assessed by flow cytometry and sFas and sFasL by ELISA within 24 h of ICU admission (Day 1), with additional measurements planned on Days 3 and 7 when feasible. The primary endpoint was 28-day all-cause mortality.

**Results:**

The manuscript reports 75 deaths among 218 patients (34.4%). At Day 1, non-survivors had higher Annexin V-positive lymphocyte percentages (28.7% ± 5.9% versus 14.3% ± 4.2%), sFas (4218 ± 863 versus 2104 ± 612 pg/mL), and sFasL (312 ± 78 versus 164 ± 43 pg/mL); the reported between-group p values were <0.001. The reported adjusted associations and combined-panel AUROC (0.891 versus 0.768 for sequential organ failure assessment) remain exploratory, in-sample estimates pending source-data audit and internal validation.

**Conclusion:**

Higher admission levels of Annexin V positivity, sFas, and sFasL were associated with 28-day mortality in the reported cohort. The apparent discrimination of the combined panel is hypothesis-generating; prospective, multicentre external validation and assessment of missing longitudinal data are required before clinical implementation or use as a therapeutic monitoring endpoint.

## 1 Introduction

Sepsis, defined as life-threatening organ dysfunction caused by a dysregulated host response to infection (Singer et al., 2016), remains one of the most significant burdens in critical care medicine worldwide. An analysis of the Global Burden of Disease Study estimated 48.9 million incident sepsis cases and 11.0 million sepsis-related deaths worldwide in 2017, accounting for approximately 19.7% of deaths in that year (Rudd et al., 2020). Despite advances in antimicrobial therapy, hemodynamic resuscitation, and organ-support technologies, mortality varies substantially across populations and case definitions (Fleischmann-Struzek et al., 2020; Evans et al., 2021).

Sepsis can involve overlapping inflammatory and immunosuppressive responses rather than a uniform sequence of two discrete phases (Hotchkiss et al., 2013; Venet and Monneret, 2018; Zhang et al., 2025; Wang et al., 2024; Pei et al., 2022). T-cell dysfunction, reduced monocyte antigen presentation, and lymphopenia have been described in critically ill patients (Hotchkiss et al., 2013; Venet and Monneret, 2018; Zhang et al., 2025; Wang et al., 2024). Apoptotic depletion of lymphocytes, including B cells and CD4-positive T cells, has been documented in human sepsis (Hotchkiss et al., 2001). Immune-cell counts and the neutrophil-to-lymphocyte ratio have also been investigated as prognostic indicators in intra-abdominal sepsis (Liu et al., 2021).

Apoptosis can involve intrinsic mitochondrial signalling and extrinsic death-receptor signalling (Elmore, 2007; Chang et al., 2007; Hotchkiss and Nicholson, 2006). Binding of Fas ligand (FasL, CD95L) to cell-surface Fas (CD95) can initiate caspase-8 signalling (Nagata, 1997). Annexin V binds externalized phosphatidylserine on the cell membrane and, together with propidium iodide exclusion, can identify an early apoptotic phenotype; this readout is not specific to Fas/FasL activation (Koopman et al., 1994). Circulating sFas and sFasL are related to the Fas pathway, but their net activities depend on molecular form and biological context; circulating levels alone do not establish pathway activation or prove a causal mechanism (Suda and Nagata, 1994; Tanaka et al., 1995; Muraki, 2022).

Clinical research provides a rationale for examining these markers. Lorente et al., (2023) reported associations of sFas levels on Days 1, 4, and 8 with 30-day mortality in an observational sepsis cohort after adjustment for SOFA. Saleki et al., (2022) examined sFasL in COVID-19 and reported that lower, not higher, sFasL was associated with severity and mortality; that direction cannot be assumed to generalize to sepsis. Jiang et al., (2018) evaluated a multicomponent lymphocyte-apoptosis model for survival in sepsis. The present study examines reported associations of three markers measured at several ICU time points, without assuming that all three specifically measure extrinsic apoptosis.

Within the broader literature on programmed cell death and experimental annexin-based interventions (Chang et al., 2007; Mui et al., 2021), we sought to describe: (1) biomarker levels at ICU admission and, where available, on Days 3 and 7; (2) associations with illness-severity measures; (3) exploratory associations with 28-day mortality; and (4) apparent discrimination and data-derived cut-offs requiring validation.

## 2 Materials and methods

### 2.1 Study design and setting

The manuscript describes a single-centre observational cohort and an electronic-record-based retrospective analysis. The stated recruitment period is January 2023 to December 2025, and the Ethics Statement names the First Affiliated Hospital of Jinzhou Medical University (approval KY11202531). Ethical approval and waiver of consent must correspond to the verified institution and collection protocol.

### 2.2 Participants

Adult patients (≥18 years) admitted to the medical-surgical ICU who met the Sepsis-3 criteria—defined as life-threatening organ dysfunction (sequential organ failure assessment [SOFA] score ≥2 from baseline) caused by a suspected or confirmed infection (Singer et al., 2016)—were eligible for inclusion. Exclusion criteria were: (1) age <18 years; (2) malignancy, haematological disease, or receipt of immunosuppressive therapy within 3 months prior to ICU admission; (3) autoimmune disease or established systemic lupus erythematosus; (4) ICU length of stay <48 h; (5) death within 24 h of ICU admission; (6) pregnancy; and (7) unavailability of Day-1 biomarker samples. The exclusions of death within 24 h and ICU stay shorter than 48 h may induce survivor-selection bias and restrict applicability to the sickest patients.

### 2.3 Data collection

Baseline clinical characteristics, comorbidities, and vital signs were abstracted from the electronic medical record at ICU admission. Severity of illness was quantified using the SOFA score and the Acute Physiology and Chronic Health Evaluation II (APACHE II) score. Laboratory parameters—including complete blood count, serum creatinine, bilirubin, lactate, procalcitonin (PCT), C-reactive protein (CRP), and coagulation panel—were extracted from the hospital laboratory information system. Microbiological culture results, primary site of infection, and antibiotic administration data were recorded. Vasopressor use, mechanical ventilation, and renal replacement therapy were recorded as organ-support interventions. Day 1 was anchored to ICU admission, not necessarily to sepsis onset or antimicrobial initiation.

### 2.4 Biomarker measurement

Peripheral blood sampling was described within 24 h of ICU admission (Day 1) and on Days 3 and 7 when clinically feasible. A reported 10-mL EDTA tube and 5-mL serum-separator tube were used. PBMCs were reportedly separated by Ficoll centrifugation; an Annexin V-FITC/propidium iodide assay (BD Biosciences) was processed within 4 h. The original description includes forward/side scatter and anti-CD45-APC/anti-CD3-PerCP-Cy5.5 staining; it is not sufficient to establish whether the analysis denominator comprised all CD45-positive lymphocytes or only CD3-positive T cells. Annexin V-positive/PI-negative events should be described as an early apoptotic phenotype within the verified parent gate rather than as a direct measurement of Fas-pathway activity. Serum sFas and sFasL were reportedly assayed by sandwich ELISA (R&D Systems).

### 2.5 Outcomes

The primary outcome was all-cause death within 28 days of ICU admission. Exploratory secondary outcomes described in the original protocol were ICU-acquired secondary infection, ICU length of stay, mechanical ventilation duration, vasopressor-free days, and SOFA scores on Days 3 and 7.

### 2.6 Statistical analysis

The original analysis plan described Shapiro–Wilk testing, t tests or Mann–Whitney tests for continuous data, chi-square or Fisher exact tests for proportions, Spearman correlations, repeated-measures ANOVA/Friedman tests for longitudinal values, stepwise logistic regression, and in-sample ROC/DeLong comparisons. These original methods and their reported results require verification rather than being presented as a completed revised analysis.

## 3 Results

### 3.1 Patient characteristics and baseline demographics

A total of 218 patients meeting eligibility criteria were included in the final analysis (see Figure 1 for the flow diagram reconstructed from reported aggregate counts). The overall 28-day mortality rate was 34.4% (75/218). As shown in Table 1, non-survivors were older (median 67 [IQR 56–74] vs. 59 [IQR 48–69] years; p = 0.003) and had higher baseline SOFA scores (10.8 ± 2.9 vs. 6.4 ± 2.1; p < 0.001) and APACHE II scores (26.3 ± 6.1 vs. 18.5 ± 5.2; p < 0.001). Pulmonary infections were the most common source of sepsis (41.7%), followed by abdominal (28.4%), urinary tract (14.7%), and bloodstream infections (15.1%). Table 1 lists septic shock in 32/75 non-survivors (42.7%) versus 32/143 survivors (22.4%); the Pearson chi-square p value calculated from these counts is 0.002, not <0.001. Mechanical ventilation was required in 69.3% of non-survivors versus 30.8% of survivors (p < 0.001). There were no statistically significant differences between groups in sex distribution or in proportions of diabetes mellitus or chronic kidney disease at baseline.

**FIGURE 1:**
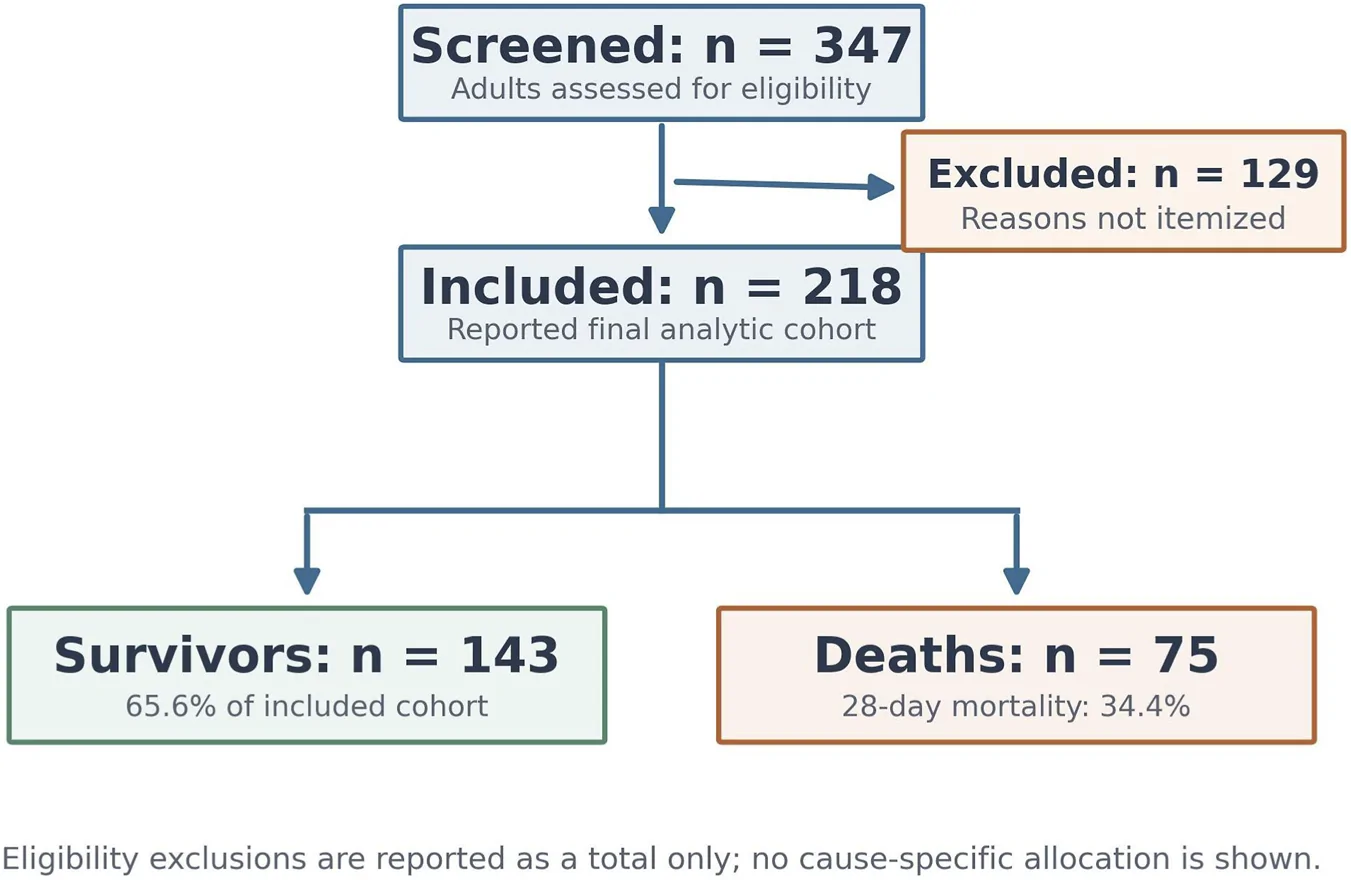
Study flow diagram based on reported aggregate counts. The manuscript reports 347 adults screened, 129 excluded and 218 included, comprising 143 survivors and 75 deaths by Day 28. Exclusion-reason counts were not supplied; the diagram does not assign counts to individual reasons.

**TABLE 1:** Baseline clinical characteristics stratified by 28-day survival status.

Characteristic	Survivors (n = 143)	Non-Survivors (n = 75)	p-value
Age, years, median (IQR)	59 (48–69)	67 (56–74)	0.003
Male sex, n (%)	85 (59.4)	46 (61.3)	0.784
BMI, kg/m^2^, mean ± SD	23.8 ± 3.4	24.2 ± 3.7	0.421
Diabetes mellitus, n (%)	43 (30.1)	26 (34.7)	0.493
Chronic kidney disease, n (%)	22 (15.4)	16 (21.3)	0.286
SOFA score (Day 1), mean ± SD	6.4 ± 2.1	10.8 ± 2.9	<0.001
APACHE II score (Day 1), mean ± SD	18.5 ± 5.2	26.3 ± 6.1	<0.001
Septic shock, n (%)	32 (22.4)	32 (42.7)	0.002
Pulmonary infection, n (%)	59 (41.3)	32 (42.7)	0.840
Abdominal infection, n (%)	42 (29.4)	20 (26.7)	0.676
Serum lactate (Day 1), mmol/L	2.1 ± 0.9	4.3 ± 1.6	<0.001
PCT, ng/mL, median (IQR)	8.4 (3.2–22.1)	24.7 (9.8–67.3)	<0.001
Mechanical ventilation, n (%)	44 (30.8)	52 (69.3)	<0.001
Vasopressor use, n (%)	38 (26.6)	47 (62.7)	<0.001
ICU length of stay, days, median (IQR)	9 (6–14)	7 (4–11)	0.021

Abbreviations: IQR, interquartile range; BMI, body mass index; SD, standard deviation; SOFA, sequential organ failure assessment; APACHE II, Acute Physiology and Chronic Health Evaluation II; PCT, procalcitonin. Septic-shock percentages and its p value have been recalculated solely from the published 2 × 2 counts; confirmation against individual records remains necessary. ICU, length of stay is truncated by death and is reported descriptively, not as reduced resource use.

### 3.2 Apoptosis biomarker levels at admission and across the first week

Table 2 displays the originally reported group summaries for admission and follow-up measurements. The admission differences are descriptive pending verification against the source dataset. Follow-up measurements were obtained only when clinically feasible; therefore the baseline group sizes (143 and 75) cannot automatically be used as Day-3 and Day-7 denominators. Death, discharge and incomplete sampling may produce informative missingness, and the original complete-case repeated-measures p values cannot be interpreted without the actual observation pattern. Figure 2 visualizes the reported means and standard deviations without assuming time-specific sample sizes or within-person trajectories.

**TABLE 2:** Reported apoptosis biomarker summaries at Days 1, 3, and 7 by 28-day outcome (mean ± SD).

Biomarker/Timepoint	Survivors (Day-1 n = 143)	Non-survivors (Day-1 n = 75)	p-value
Annexin V^+^ lymphocytes (%), Day 1	14.3 ± 4.2	28.7 ± 5.9	<0.001
Annexin V^+^ lymphocytes (%), Day 3	10.8 ± 3.6 [n VERIFY]	34.1 ± 7.2 [n VERIFY]	<0.001
Annexin V^+^ lymphocytes (%), Day 7	7.4 ± 2.9 [n VERIFY]	38.6 ± 9.1 [n VERIFY]	<0.001
sFas (pg/mL), Day 1	2104 ± 612	4218 ± 863	<0.001
sFas (pg/mL), Day 3	1876 ± 548 [n VERIFY]	4891 ± 1024 [n VERIFY]	<0.001
sFas (pg/mL), Day 7	1542 ± 493 [n VERIFY]	5137 ± 1186 [n VERIFY]	<0.001
sFasL (pg/mL), Day 1	164 ± 43	312 ± 78	<0.001
sFasL (pg/mL), Day 3	138 ± 37 [n VERIFY]	348 ± 84 [n VERIFY]	<0.001
sFasL (pg/mL), Day 7	112 ± 31 [n VERIFY]	371 ± 96 [n VERIFY]	<0.001

Abbreviations: sFas, soluble Fas; sFasL, soluble Fas ligand. Day-1 cohort totals are 143 survivors and 75 non-survivors; these are not verified time-specific denominators for Days 3 and 7. The displayed means, SDs, and nominal p values at follow-up are reproduced from the original manuscript, not independently reanalysed.

**FIGURE 2:**
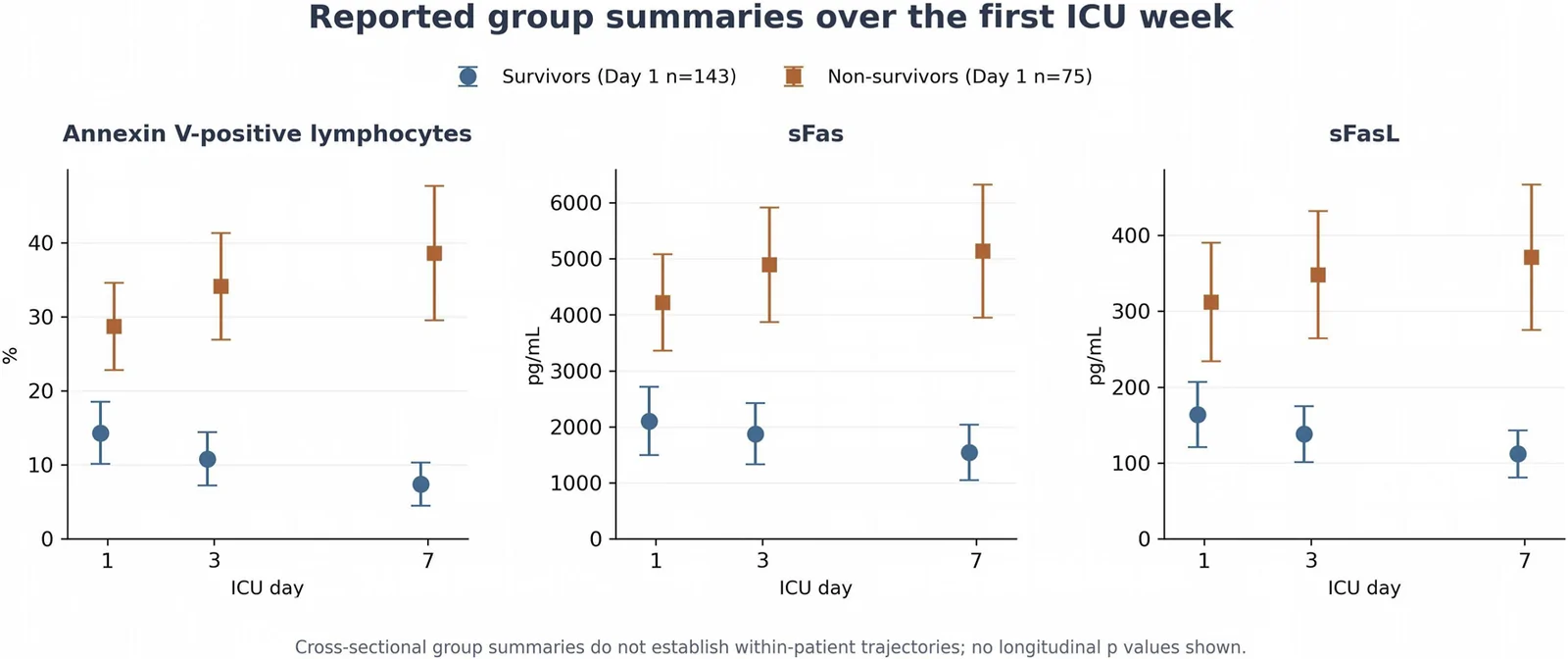
Reported apoptosis biomarker means and standard deviations on ICU Days 1, 3 and 7. Points show the group means and whiskers show the standard deviations reproduced from Table 2. Day-1 denominators were 143 survivors and 75 non-survivors; Day-3 and Day-7 denominators and missingness were not supplied. These aggregate cross-sections are not longitudinal patient trajectories; inferential temporal statistics are not recalculated.

### 3.3 Correlation with organ dysfunction severity

Table 3 summarises Spearman correlation coefficients between Day-1 apoptosis biomarkers and indices of disease severity. All three biomarkers correlated significantly and positively with Day-1 SOFA score (Annexin V^+^%: r = 0.61; sFas: r = 0.58; sFasL: r = 0.49; all p < 0.001) and with APACHE II score (r = 0.54, 0.52, and 0.43 respectively; all p < 0.001). Moderate correlations were observed with serum lactate, PCT, and absolute lymphocyte count. Notably, Annexin V^+^ percentage correlated inversely with absolute lymphocyte count (r = −0.48; p < 0.001), compatible with, but not proof of, a link between an early apoptotic phenotype and lower circulating lymphocyte counts. The 21 reported correlation tests are exploratory; interpretation should account for multiple comparisons.

**TABLE 3:** Spearman correlation coefficients between Day-1 apoptosis biomarkers and clinical severity parameters.

Parameter	Annexin V^+^% (Day 1)	sFas (Day 1)	sFasL (Day 1)
SOFA score (Day 1)	r = 0.61, p < 0.001	r = 0.58, p < 0.001	r = 0.49, p < 0.001
APACHE II score (Day 1)	r = 0.54, p < 0.001	r = 0.52, p < 0.001	r = 0.43, p < 0.001
Serum lactate (Day 1)	r = 0.47, p < 0.001	r = 0.44, p < 0.001	r = 0.38, p < 0.001
Procalcitonin (Day 1)	r = 0.39, p < 0.001	r = 0.36, p < 0.001	r = 0.31, p = 0.002
Absolute lymphocyte count	r = −0.48, p < 0.001	r = −0.41, p < 0.001	r = −0.34, p < 0.001
C-reactive protein	r = 0.32, p < 0.001	r = 0.29, p < 0.001	r = 0.27, p = 0.006
Serum creatinine	r = 0.28, p = 0.003	r = 0.31, p < 0.001	r = 0.24, p = 0.011

Abbreviations: SOFA, sequential organ failure assessment; APACHE II, Acute Physiology and Chronic Health Evaluation II; sFas, soluble Fas; sFasL, soluble Fas Ligand. Correlation p values are nominal and exploratory. Several displayed r/p combinations (e.g., r = 0.24, p = 0.011) disagree with the usual n = 218 correlation-test approximation (approximately p = 0.00035); confirm the actual pairwise sample sizes, ties, test method, p values, and multiplicity strategy using the patient-level dataset.

### 3.4 Univariate and multivariate predictors of 28-day mortality

Table 4 presents the originally reported univariate and adjusted associations between admission biomarkers, clinical variables, and 28-day mortality, except that the crude vasopressor OR and 95% CI have been recalculated from Table 1 counts. With 75 deaths, the number of candidate parameters and correlations between severity measures raise concerns about overfitting. The original stepwise selection procedure, model coefficients/intercept, multicollinearity diagnostics and internal validation are not provided. Thus adjusted odds ratios must be regarded as unverified exploratory estimates rather than validated independent predictors. For interpretability, the reported rounded sFas adjusted OR of 1.002 per 1 pg/mL corresponds algebraically to approximately 1.22 per 100 pg/mL (the CI obtained by rescaling its rounded endpoints is approximately 1.11–1.49); the reported sFasL adjusted OR of 1.014 per 1 pg/mL corresponds to approximately 1.15 per 10 pg/mL (rounded-endpoint CI approximately 1.07–1.24). These unit conversions are not refitted estimates.

**TABLE 4:** Univariate and multivariate logistic regression for 28-day mortality.

Variable	Univariate OR (95% CI)	Univariate p	Adjusted OR (95% CI)	Adjusted p
Annexin V^+^% (Day 1, per 1%)	1.22 (1.14–1.31)	<0.001	1.18 (1.09–1.28)	<0.001
sFas (Day 1, per pg/mL)	1.003 (1.002–1.004)	<0.001	1.002 (1.001–1.004)	0.001
sFasL (Day 1, per pg/mL)	1.018 (1.010–1.026)	<0.001	1.014 (1.007–1.022)	<0.001
SOFA score (Day 1)	1.48 (1.31–1.68)	<0.001	1.35 (1.18–1.55)	<0.001
APACHE II score (Day 1)	1.16 (1.10–1.22)	<0.001	1.09 (1.02–1.16)	0.008
Serum lactate (Day 1)	1.62 (1.32–1.98)	<0.001	1.38 (1.10–1.74)	0.006
Vasopressor use	4.64 (2.55–8.43)	<0.001	2.74 (1.33–5.65)	0.006
PCT (≥1 ng/mL)	2.31 (1.18–4.52)	0.014	1.47 (0.69–3.12)	0.317
Age (per year)	1.03 (1.01–1.06)	0.012	1.02 (0.99–1.05)	0.211

The displayed adjusted results are those originally reported; the table contains adjusted estimates for age and PCT, even though the original footnote lists only four covariates.

### 3.5 Diagnostic performance and optimal cut-off values

The original manuscript reports apparent Day-1 AUROCs of 0.843 for Annexin V positivity, 0.827 for sFas, 0.791 for sFasL, 0.768 for SOFA, 0.779 for APACHE II and 0.891 for a combined biomarker/SOFA model. The combined estimate and its DeLong comparison (originally reported p = 0.003 versus SOFA) were calculated in the same development cohort; they are not optimism-corrected or externally validated. Data-derived cut-offs are exploratory. The original PPV/NPV entries in Table 5 are inconsistent with the reported sensitivity, specificity and group sizes; the edited values are arithmetic implications of the nearest integer 2 × 2 confusion matrices, not independently verified predictions. Figure 3 displays the reported AUROC point estimates and 95% confidence intervals; full ROC curves cannot be reconstructed from these summaries alone.

**TABLE 5:** Receiver operating characteristic (ROC) analysis for 28-day mortality prediction.

Predictor	AUROC (95% CI)	Cut-off	Sensitivity (%)	Specificity (%)	PPV (%)	NPV (%)
Annexin V^+^% (Day 1)	0.843 (0.789–0.897)	20.5%	80.0	83.2	71.4*	88.8*
sFas (Day 1)	0.827 (0.773–0.882)	3,180 pg/mL	76.0	84.6	72.2	87.1*
sFasL (Day 1)	0.791 (0.730–0.852)	238 pg/mL	72.0	81.1	66.7*	84.7*
SOFA score (Day 1)	0.768 (0.703–0.834)	≥8	74.7	72.0	58.3*	84.4*
APACHE II (Day 1)	0.779 (0.717–0.842)	≥23	72.0	76.9	62.1*	84.0*
Combined panel^†^	0.891 (0.846–0.937)	N/A	85.3	86.7	77.1*	91.9*

^†^ The original combined panel was described as Annexin V positivity + sFas + sFasL + SOFA (logistic-regression probability). The model coefficients, intercept and operating threshold were not supplied; the reported AUROC 0.891 and DeLong p = 0.003 are unvalidated apparent results. The asterisk-marked PPV/NPV estimates were calculated from approximate integer confusion matrices compatible with the reported sensitivity/specificity and 75 deaths and 143 survivors. These estimates require verification against the underlying patient-level predictions. Abbreviations: AUROC, area under the ROC curve; PPV, positive predictive value; NPV, negative predictive value.

**FIGURE 3:**
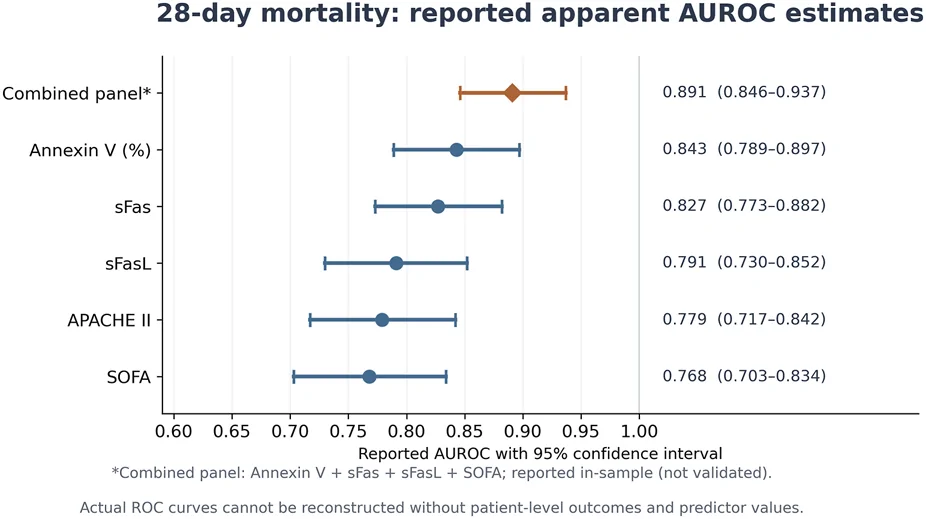
Reported apparent AUROC estimates and 95% confidence intervals. The figure displays the six AUROC estimates from Table 5 as a confidence-interval plot, not as ROC curves. The combined-panel model is an unvalidated in-sample estimate. ROC coordinates cannot be recovered from AUROC summary statistics; patient-level outcomes and predictor scores are required.

### 3.6 Subgroup analyses

The original manuscript reports infection-source subgroup interactions (Annexin V positivity p = 0.41; sFas p = 0.38), but prespecified subgroup definitions and sample sizes were not supplied. For consistency with Table 1, the septic-shock subgroup comprises 64 patients (32 survivors and 32 non-survivors); the conflicting previously reported subgroup count is not used, and its estimate is withheld pending source-data verification. In addition, 58 ICU-acquired secondary infections (26.6%) were reported, with higher Day-3 Annexin V positivity and sFas in patients classified as having secondary infection; infection-onset timing, clinical case definition, Day-3 eligibility and adjustment for confounding were not supplied. These associations must not be interpreted as evidence that apoptosis causes infection.

### 3.7 Descriptive figure summaries and unavailable source data

Figure 4 diagrams the proposed Day-1 biomarker strata but does not present Kaplan–Meier curves, because subgroup membership, event times and censoring data were not provided. Figure 5 provides a heatmap of the reported admission means by 28-day outcome; the originally described SOFA-stratified heatmap cannot be reconstructed because the SOFA categories and within-stratum values are unavailable. These two figures are explicitly descriptive substitutes, not reconstructed patient-level analyses.

**FIGURE 4:**
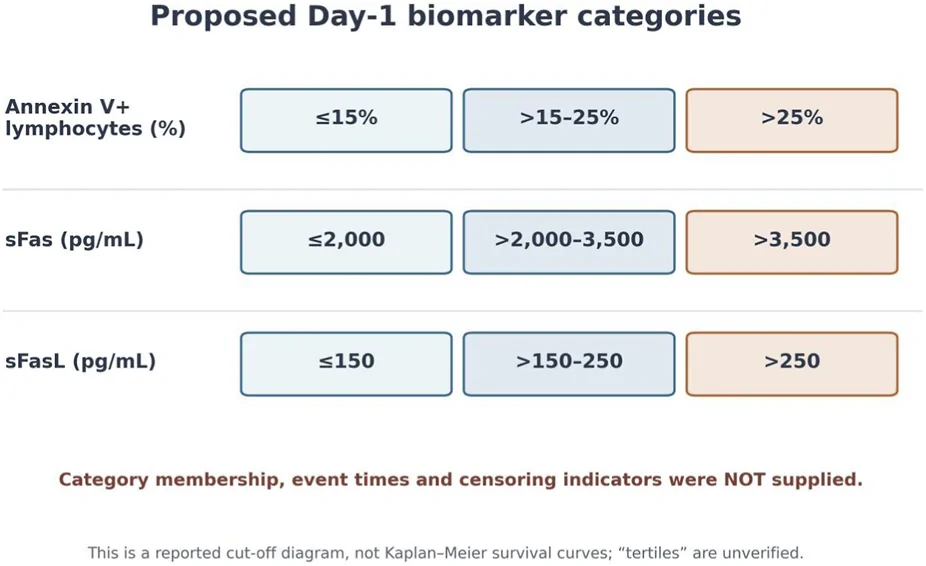
Proposed Day-1 biomarker stratification thresholds (schematic; not Kaplan–Meier curves). Proposed categories are Annexin V positivity (≤15%, >15–25%, >25%); sFas (≤2,000, >2,000–3,500, >3,500 pg/mL); and sFasL (≤150, >150–250, >250 pg/mL). Patient category membership, survival times and censoring were not supplied, so Kaplan–Meier survival curves and equal-size tertiles cannot be verified or reconstructed.

**FIGURE 5:**
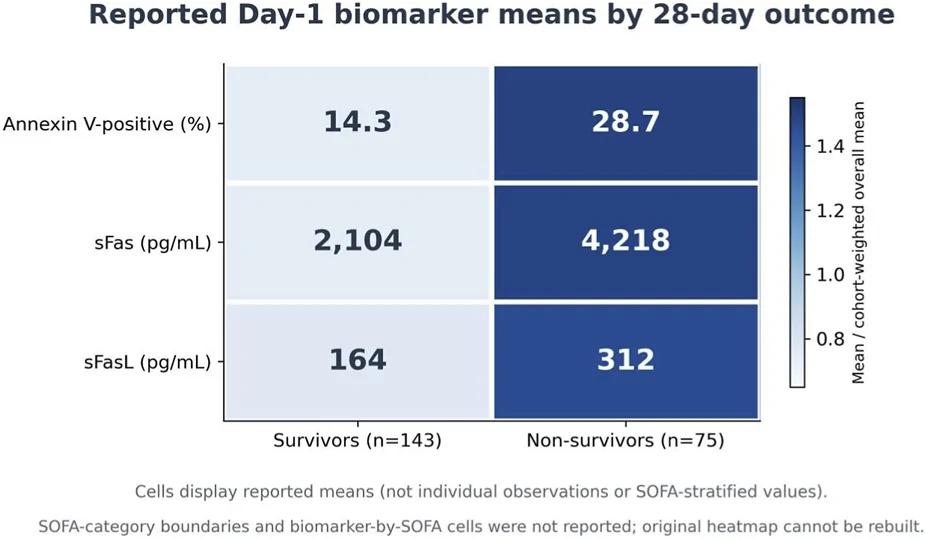
Heatmap of reported Day-1 biomarker means by 28-day outcome (not SOFA-stratified). Cells show Table 2 admission means in the original units. Colour represents each mean divided by its cohort-weighted overall mean using reported baseline group sizes of 143 and 75. The originally proposed heatmap by SOFA category cannot be reconstructed without the category definitions and cross-stratified biomarker values.

## 4 Discussion

In this reported single-centre cohort, higher baseline Annexin V positivity, sFas and sFasL were associated with subsequent mortality in the original analyses. Annexin V reports a general early apoptotic phenotype rather than Fas-specific signalling. The reported combined AUROC of 0.891 is an apparent in-sample result and has not been internally or externally validated. Original later-timepoint trends are subject to unquantified missingness, and the Day-3 secondary-infection association is vulnerable to reverse temporality and confounding. Thus the findings are exploratory and do not establish causality or clinical utility.

Lorente et al., (2023) studied serum sFas on Days 1, 4 and 8 and reported associations with 30-day mortality after adjustment for SOFA, providing relevant observational context. Another observational study examined soluble Fas following major trauma in relation to delayed neutrophil apoptosis and subsequent sepsis (Paunel-Görgülü et al., 2011); that study population and outcome are distinct from the present cohort. The biological interpretation of sFas and sFasL is complex: soluble Fas may bind ligand, whereas soluble FasL activity depends on cleavage state and molecular organization (Suda and Nagata, 1994; Tanaka et al., 1995; Muraki, 2022). Circulating concentrations do not reveal whether measured analytes reflect increased production, shedding, clearance or functional death-receptor signalling. Notably, Saleki et al. (2022) reported lower sFasL in more severe COVID-19, illustrating that the direction of association may differ across diseases. The present observational results should not be used to assert that sFasL directly kills lymphocytes or that sFas is a proven compensatory response.

The original manuscript reports a higher apparent Day-1 AUROC for Annexin V positivity than for the other two markers, but comparison of their performance and any mechanistic interpretation require source-data verification. Jiang et al. (2018) investigated a lymphocyte-apoptosis prediction model in sepsis, while Annexin V binding reflects phosphatidylserine externalization in several apoptotic pathways (Chang et al., 2007; Koopman et al., 1994). The flow-cytometric parent population and preanalytical consistency also require clarification; feasibility and reproducibility in routine clinical practice cannot be established from the current reporting (Greineder et al., 2007).

An inverse reported association between Annexin V positivity and lymphocyte count and positive associations with SOFA are compatible with previous observations of immune dysregulation in sepsis (Hotchkiss et al., 2013; Venet and Monneret, 2018; Hotchkiss et al., 2001), but they do not identify causal mechanisms. Other processes, including altered trafficking and proliferation, may influence circulating lymphocyte counts (Wang et al., 2024). Reviews of annexin-based interventions describe experimental therapeutic mechanisms (Mui et al., 2021); this observational biomarker study does not establish that Annexin V-defined apoptosis is a validated pharmacodynamic endpoint or that therapy guided by these measurements improves clinical outcomes.

Several limitations deserve explicit emphasis. First, the actual study centre, observation period, specimen provenance and temporal relationship between sample collection and retrospective analysis must be verified. Excluding patients dying within 24 h and those with ICU stays below 48 h can introduce survivor-selection bias; excluded patients' characteristics are unavailable in the submitted material. Second, missing measurements at Days 3 and 7, death before sampling and early discharge may cause informative attrition, making complete-case trajectories unreliable. Third, only 75 deaths were available for numerous correlated candidate predictors, with no complete model equation, internal validation, calibration intercept/slope, Brier score or external validation. Fourth, the original biomarker distributions, instrument/assay QC, lymphocyte-versus-CD3 gate, timing relative to sepsis recognition, and exact secondary-infection definition were insufficiently documented. Fifth, multiple exploratory comparisons may inflate false-positive findings, and cut-offs selected in the same cohort are likely optimistic. Finally, shorter ICU stays among non-survivors are mechanically truncated by death and cannot be equated with faster recovery or reduced resource utilization. Prospective multicentre validation with a prespecified analysis, documented sample availability and competing-risk-aware outcome methods is needed.

## 5 Conclusion

In this reported cohort, admission Annexin V positivity, serum sFas and sFasL were associated with 28-day mortality, and the originally reported combined model showed promising apparent discrimination. However, missing longitudinal observations, selection bias, incomplete laboratory and modelling documentation, and the absence of internal or external validation preclude claims of established incremental clinical benefit. These exploratory findings require verification of the original data and prospective multicentre validation before any recommendation for routine risk stratification or therapeutic monitoring.

## Data Availability

The summary data supporting the reported findings are included in the article. Further inquiries can be directed to the corresponding author.
